# Forecasting Financial Time Series through Causal and Dilated Convolutional Neural Networks

**DOI:** 10.3390/e22101094

**Published:** 2020-09-29

**Authors:** Lukas Börjesson, Martin Singull

**Affiliations:** Department of Mathematics, Linköping University, 581 83 Linköping, Sweden; lukbo072@student.liu.se

**Keywords:** deep learning, financial time series, causal and dilated convolutional neural networks

## Abstract

In this paper, predictions of future price movements of a major American stock index were made by analyzing past movements of the same and other correlated indices. A model that has shown very good results in audio and speech generation was modified to suit the analysis of financial data and was then compared to a base model, restricted by assumptions made for an efficient market. The performance of any model, trained by looking at past observations, is heavily influenced by how the division of the data into train, validation and test sets is made. This is further exaggerated by the temporal structure of the financial data, which means that the causal relationship between the predictors and the response is dependent on time. The complexity of the financial system further increases the struggle to make accurate predictions, but the model suggested here was still able to outperform the naive base model by more than 20% and 37%, respectively, when predicting the next day’s closing price and the next day’s trend.

## 1. Introduction

Deep learning has brought a new paradigm into machine learning in the past decade and has shown remarkable results in areas such as computer vision, speech recognition and natural language processing. However, one of the areas where it is yet to become a mainstream tool is in forecasting financial time series. This despite the fact that time series does provide a suitable data representation for deep learning methods such as a convolutional neural network (CNN) [1]. Researchers and market participants (Market participants is a general expression for individuals or groups who are active in the market, such as banks, investors, investment funds, or traders (for their own account); often, we use the term *trader* as a synonym for *market participant* [2]) are still, to the most part, sticking to more historically well known and tested approaches, but there has been a slight shift of interest to deep learning methods in the past years [3]. The reason behind the shift, apart from the structure of the time series, is that the financial market is an increasingly complex system. This means that there is a need for more advanced models, such as deep neural networks, that do a better job in finding the nonlinear relations in the data.

Omar Berat Sezer et al. [3] gives a very informative review of the published literature on the subject between 2005 and 2019 and states that there has been a trend towards more usage of deep learning methods in the past five years. The review covers a wide range of deep learning methods, applied to various time series such as stock market indices, commodities and forex. From the review, it is clear that CNNs is not the topmost used method and that developers have focused more on recurrent neural networks (RNNs) and long short-term memory (LSTM) networks. The CNNs are, however, very good at building up high-level features from lower-level features that were originally found in the input data, which is not something a LSTM network is primarily designed to do.

However, the CNNs and LSTM networks do not need to be used as two separate models, but they could be used as two separate parts of the same network. An example is to use a CNN to preprocess the data, in order to extract suitable features, which could then be used as the input to the LSTM part of the network [4].

Furthermore, the WaveNet structure considered in [5] suggests that the model can catch long-and short-term dependencies, which is what the LSTM is designed to do as well. Although this is something that will not be further explored in this paper, it does provide further research questions, such as if the WaveNet can be used as a sort of preprocessing to a LSTM network. Another example would be to process the data through a CNN and a LSTM separately and then combine them, before the final output, in a suitable manner. This is something that is explored, with satisfactory results, in [6], and the CNN part of the network is in fact an implementation of the WaveNet here as well. However, only the LSTM part of the network handles the exogenous series; therefore, for future work, it would be interesting to see if the performance could be improved by making the WaveNet handle the exogenous series as well.

Papers where the WaveNet composes the whole network instead of just being a component in one exist as well. Two examples are [7,8], and these models take into consideration exogenous series as well. However, they used ReLU as the activation function instead of SeLU, but they implemented normalization of the network in a similar way as will be done in this paper. Furthermore, when considering the exogenous series, their approach regarding the output from each residual layer was different. Instead of extracting the residual from each exogenous series, which will be done in this paper, only the combined residual was used. See Section 3.2 for more details.

In contrast to the approach taken in this paper, and by all who try to fit statistical models on financial time series, there are those who state that complexity is not the issue, but instead advocate for the Efficient Market Hypothesis (EMH) [9]. A theory that essentially suggests that no model, no matter how complex, can outperform the market, since the price is based on all available information. The theory rests upon three key assumptions—(1) no transaction costs, (2) cost is not a barrier to obtain available information and (3) all market participants agree on the implications of the available information—which are stated to be sufficient, but not necessary (sufficient but not necessary means, in this context, that the assumptions do not need to be fulfilled at all times; for example, the second assumption might be loosened from including all to only a sufficient number of traders). These assumptions, even with modifications, are very bold, and there are many who have criticized the theory over the years. However, whether one agrees with the theory or not, one would probably agree with the statement that a model which satisfies the assumptions made in EMH would indeed be suitable as a base model. This means that such a model can be used as a benchmark in order to assess the accuracy of other models.

Traders and researchers alike would furthermore agree that the price of any asset is, apart from its inner utility, based on the expectation of its future value. For example, the price of a stock is partially determined by the company’s current financials, but also by the expectation of future revenues or future dividends. This expectation is, by the neoclassical economics, seen as completely rational, giving rise to the area of rational expectation [10]. However, the emergence of behavioral economics has questioned this rationality and proposes that traders (or more generally, decision-makers who act under risk and uncertainty) are sometimes irrational and many times affected by biases [11].

A trader that sets out to exploit this irrationality and these biases can only do so by looking into the past and, thereby, also go against the hypothesis of the efficient market. Upon reading this, it should be fairly clear that making predictions in the financial markets is no trivial task, and it should be approached with humility. However, one should not be discouraged since the models proposed in [3] do provide promising or, oftentimes, positive results.

An important note about the expectation mentioned above is that the definition of a trader, provided by Paul and Baschnagel, is not limited to a human being; it might as well be an algorithm. This is important since the majority of transactions in the market are now made by algorithms. These algorithms are used in order to decrease the impact of biases and irrationality in decision making. However, the algorithms are programmed by people and are still making predictions under uncertainty, based on historical data, which means that they are by no means free of biases. Algorithms are also more prone to get stuck in a feedback loop, which has been exploited by traders in the past. An interesting example is the two Norwegians, Svend Egil Larsen and Peder Veiby, who in 2010 were accused of manipulating algorithmic trading systems. They were, however, acquitted in 2012, since the court found no wrongdoing.

The aim of this paper is to expand the research in forecasting financial time series, specifically with a deep learning approach. To achieve this, two models, which greatly differ in the approach towards the effectiveness of the market, are compared. The first model is restricted by the assumptions made on the market by the EMH and is seen as the base model. The second model is a CNN, inspired by the WaveNet structure [5], and is influenced by a model developed for audio and speech generation by researchers at Google. The models set out to predict the next day’s closing price as well as the trend (either up or down) of Standard and Poor’s 500 (S&P 500), a well-known stock market index comprising 500 large companies in the US.

The outline of this paper is as follows. In Section 2 we give a brief background to the theory needed and in Section 3 formulate the considered models and discuss the methodology. The results of the study are given in Section 4 with a discussion in Section 5. We conclude the paper in Section 6.

## 2. Theoretical Background

### 2.1. Time Series

When using time series as a forecasting model, one makes the assumption that future events, such as the next day’s closing price of a stock, can be determined by looking at past closing prices in the time series. Most models, however, include a random error as a factor, meaning that there is some noise in the data which cannot be explained by past values in the series.

Furthermore, the models can be categorized as parametric or non-parametric, where the parametric models are the ones most regularly used. In the parametric approach, each data point in the series is accompanied by a coefficient, which determines the impact the past value has on the forecast of future values in the series.

The linear autoregressive model of order *p*, written as AR(p), is given by

(1) $$X_{t}=c+\sum_{i=1}^{p}\varphi_{i}X_{t-i}+\varepsilon_{t},$$

with unknown parameters $\varphi_{i}$ and $\varepsilon_{t}$ as white noise. This is one of the most well known time series models, and it is a model where the variable is regressed against itself (auto meaning “oneself”, when used as a prefix). It is often used as a building block to more advanced time series models, such as the autoregressive moving average (ARMA) or the generalized autoregressive conditional heteroskedasticity (GARCH) models. However, the AR process will not be considered as a building block in the models proposed in this paper. Instead, the proposed CNN models in this paper can be represented as the nonlinear version of the AR model, or NAR(p) for short, given as

(2) $$X_{t}=c+f\left(X_{t-1},\ldots ,X_{t-p}\right)+\varepsilon_{t},$$

with a nonlinear function f(·,…,·). In fact, a large number of machine learning models, when applied to time series, can be seen as AR or NAR models. This might seem obvious to some, but it is something that is seldom mentioned in the scientific literature. Furthermore, the models can be generalized to a nonlinear autoregressive exogenous (NARX) model. Given $Z_{k}$ as an exogenous time series and $\psi_{k}$ its accompanied coefficient, we have the NARX(p,r) model

(3) $$X_{t}=c+f\left(X_{t-1},\ldots ,X_{t-p},Z_{t-1},\ldots ,Z_{t-r}\right)+\varepsilon_{t}.$$

When determining the coefficients in the autoregressive models, most models need the underlying stochastic processes $\left\{X_{t}:t\geq 0\right\}$ to be stationary or at least weak-sense stationary. This means that we are assuming that the mean and the variance of $X_{t}$ are constant over time. However, when looking at historical prices in the stock market, one can clearly see that this is not the case, for either the variance or the mean. All of the above models can be generalized to handle this non-stationarity by applying suitable transformations to the series. These transformations, or integrations, are a necessity when determining the values for the coefficients, for most of the well-known methods, although this need not be the case when using a neural network [12].

### 2.2. Neural Networks

The neural network, when applied on a supervised problem, sets out to minimize a certain predefined loss function. The loss function used in this paper, when calculating the next day’s closing price, was the mean absolute percentage error (MAPE)

$$\epsilon \left(w\right)=\frac{100}{n}\sum_{i=1}^{n}\left|\frac{y_{i}-t_{i}}{t_{i}}\right|,$$

where $t_{i}$ is the *i*th target value and $y_{i}$ is the model’s prediction. The reason for this is that the errors are now made proportional with respect to the target value. This is important, since the mean and variance of financial series cannot be assumed to be stationary, and this would otherwise skew the accuracy of the model, unproportionally, to times characterized by a low mean. Meanwhile, the loss function used for classifying the next day’s trend was the binary crossentropy

$$\epsilon \left(w\right)=\frac{1}{n}\sum_{i=1}^{n}t_{i}\log\left(y_{i}\right)+\left(1-t_{i}\right)\log\left(1-y_{i}\right)).$$

The loss function is with respect to the weight w, and the loss is minimized when choosing the weights that solve the function

$$\frac{\partial \epsilon (w)}{\partial w}=0.$$

However, this algebraic solution is seldom achievable, and numerical solutions are more often used. These numerical methods set out to find points in close proximity to a local (hopefully global, but probably not) optima.

Moreover, instead of calculating the gradient with respect to each weight individually, backpropagation uses the chain rule, where each derivative can be computed layerwise backward. This leads to a decrease in complexity, which is very important, since it is not unusual that the number of weights might be counted in thousands or in tens of thousands.

The neural network is, unless stated otherwise, considered to be a fully connected network, which means that all weights, in two adjacent layers, are connected to each other. Although backpropagation did a remarkable job in decreasing the complexity, the fully connected models are not good at scaling to many hidden layers. This problem can be solved by having a sparse network, which means that not all weights are connected. The CNN model, further explained in the next section, is an example of a sparse network, where not all units are connected and where some units also share weights.

### 2.3. Convolutional Neural Networks

The input to the CNN, when modeling time series, is a three-dimensional tensor, i.e., (nr of observations)×(width of the input)×(nr of series). The number of series is here the main series, for which the predictions will be made over, plus optional exogenous series.

Furthermore, in the CNN model, there is an array of hyperparameters that defines the structure and complexity of the network. Below is a short explanation of the most important parameters to be acquainted with in order to understand the networks proposed in this paper.

#### 2.3.1. Activation Function

In its simplest form, when it only takes on binary values, the activation function determines if the artificial neuron fires or not. More complex activation functions are often used, and the sigmoid and tanh functions

(4) $$\begin{array}{cc} g(x) & =\frac{e^{x}}{e^{x}+1},\\ g(x) & =tanh(x), \end{array}$$

are two examples, which have been used to a large extent in the past. They are furthermore two good examples of activation functions that can cause the problem of vanishing gradients (studied by Sepp Hochreiter in 1991, but further analyzed in 1997 by Sepp Hochreiter and Jürgen Schmidhuber [13]), which of course is something that should be avoided. A function that does not exhibit this problem is the rectified linear unit (ReLU) function

$$g\left(x\right)=\left\{\begin{array}{cc} 0 & \mathrm{if}\;x\leq 0,\\ x & \mathrm{otherwise}, \end{array}\right.$$

which has gained a lot of traction in recent years, and is today the most popular one for deep neural networks. One could easily understand why ReLU avoids the vanishing gradient problem, by looking at its derivative

$$g^{\prime }\left(x\right)=\left\{\begin{array}{cc} 0 & \mathrm{if}\;x\leq 0,\\ 1 & \mathrm{otherwise}, \end{array}\right.$$

and from it conclude that the gradient is either equal to 0 or 1. However, the derivative also shows a different problem that comes with the ReLU function, which is that the gradient might equal zero, and that the output from many of the nodes might in turn become zero. This problem is called the dead ReLU problem, and it might cause many of the nodes to have zero impact on the output. This can be solved by imposing minor modifications on the function, and it therefore now comes in an array of different flavors. One such flavor is the exponential linear unit (ELU)

$$g\left(x\right)=\left\{\begin{array}{cc} \alpha (e^{x}-1) & \mathrm{if}\;x\leq 0,\\ x & \mathrm{otherwise}, \end{array}\right.$$

where the value of alpha is often chosen to be between 0.1 and 0.3. The ELU solves the dead ReLU problem, but it comes with a greater computational cost. A variant of the ELU is the scaled exponential linear unit (SELU)

(5) $$g\left(x\right)=\lambda \left\{\begin{array}{cc} \alpha e^{x} & \mathrm{if}\;x\leq 0,\\ x & \mathrm{otherwise}, \end{array}\right.$$

which is a relatively new activation function, first proposed in 2017 [14]. The values of α and λ have been predefined by the authors, and the activation also needs the weights in the network to be initialized in a certain way, called lecun_normal. lecun_normal initialization means that the start value for each weight is drawn from a standard normal distribution.

Normalization can be used as a preprocessing of the data, due to its often positive effect on the model’s accuracy, and some networks also implement batch normalization at some point or points inside the network. This is what is called external normalization. However, the beauty of SeLU is that the output of each node is normalized, and this process is fittingly called internal normalization. Internal normalization proved to be more useful than external normalization for the models in this paper, which is why SeLU was used throughout the network in the final models.

#### 2.3.2. Learning Rate

The learning rate, often denoted by η, plays a large role during the training phase of the models. After each iteration, the weights are updated by a predefined update rule such as gradient descent

$$w_{i+1}=w_{i}-\eta \nabla \epsilon \left(w_{i}\right),$$

where $\nabla \epsilon (w_{t})$ is the gradient for the loss function at the *i*th iteration. The learning rate, η, can here be seen as determining the rate of change in every iteration. Gradient descent is but one of many update rules, or optimizers (as they are more often called), and it is by far one of the simplest. More advanced optimizers are often used, such as the adaptive moment estimation (Adam) [15], which has, as one if perks, individual learning rates for each weight. The discussion about optimizers will not continue further in this paper, but it should be clear that the value of the learning rate and the choice of optimizer have a great impact on the overall performance of the model.

#### 2.3.3. Filters

**Figure 1:**
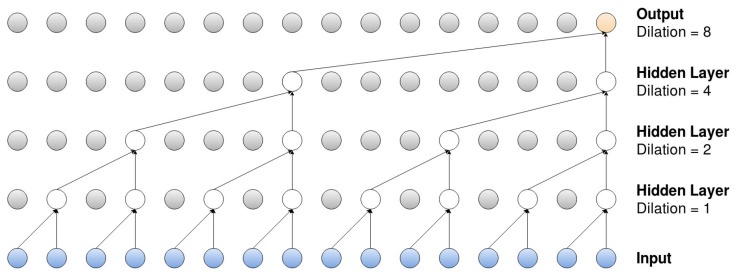
Dilated convolutional layers for an input series of length 16.

The filter dimensions need to be determined before training the model, and the appropriate dimensions depend on the underlying data and the model of choice. When analyzing time series, the filter needs to be one dimensional, since the time series is just an ordered sequence. The developer needs to determine just two things: the width of the filters (Figure 1 shows a filter with the width equal to two) and then how many filters to use for each convolutional layer. The types of features that the convolutional layer “searches” for are highly influenced by the filter dimensions, and having multiple filters means that the network can search for more features in each layer.

#### 2.3.4. Dilation

A dilated convolutional filter is a filter that, not surprisingly, is widened but still uses the same number of parameters. The filter is widened by neglecting certain inputs, and an example of this can be observed in Figure 1. The bottom layer represents the input, in the form of a time series $x=(x_{1},x_{2},\ldots ,x_{n})$ (for some time *n*, onto which repeated dilated convolutions, with increasing dilation rates, are applied; the filter width is again set to equal two in the observed model). The first hidden layer applies dilated convolutions with the dilation rate equal to one, meaning that the layer applies the filter onto two adjacent elements, $x_{i}$ and $x_{i+1}$, of the input series. The second layer applies dilated convolutions, with the rate now set to equal two, which means that the filter is applied onto elements $x_{i}$ and $x_{i+2}$ (notice here that the number of parameters remains the same, but the filter width has been “widened”). Lastly, the third and fourth layer have rates equal to four and eight, so the filter is applied onto elements $x_{i}$ and $x_{i+4}$, and $x_{i}$ and $x_{i+8}$, respectively.

#### 2.3.5. Dropout

The dropout factor is a way to prevent the model from overfitting to the training data, and it does this by setting a fraction of the weights in a certain layer to zero. This leads to a decrease in complexity, but the developer does not have control over which nodes will be set to zero (i.e., the weights are chosen at random). Hence, dropout is not the same as changing the number of nodes in the network. For more details, see [16].

### 2.4. WaveNet

**Figure 2:**
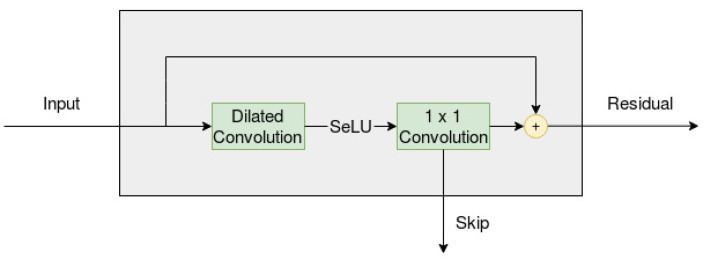
Overview of the residual layer, when only the main series is used as input.

The CNN models proposed in this paper are inspired by the WaveNet structure, modeled by van den Oord et al. in 2016 [5]. The main part of the network in a WaveNet can be visualized in Figure 2, which incorporates a dilated (and causal) convolution and a 1×1 convolution (i.e., the width of the filter set to equal one). The input from the left side is the result of a casual convolution, with filter size equal to two, which has been applied to the original input series as a sort of preprocessing. The output on the right side of the layer is the residual, which can be used as the input to a new layer, with an identical set up. The number of residual connections must be predetermined by the developer, but the dilated convolution also sets an upper limit on how many connections can be used. Figure 1 displays repeated dilations on an input series with length equal to 16, and we can see that the number of layers has an upper limit of four.

Furthermore, the output from the bottom of each layer is the skip, which is the output that is passed on to the following layers in the network. If four layers are used, as in Figure 1, then the network would end up with four skip connections. These skip connections are then added (element-wise) together to form a single output series. This series is then passed through two 1×1 convolutions, and the result of this will be the output of the model.

The WaveNet has three important characteristics: it is dilated, causal and has residual connections. This means that the network is sparsely connected, that calculations can only include previous values in the input series (which can be observed in Figure 1) and that information is preserved across multiple layers. The sparsity is also further increased by having the width of the filters equal to only either one or two.

The WaveNet sets out to maximize the joint probability of the series $x={\left(x_{t},\ldots ,x_{t-p}\right)}^{T}$, for any time *t* and length equal to *p*, which is factorized as a product of conditional probabilities

$$p\left(x\right)=\prod_{i=1}^{t}p\left(x_{i}|x_{1},\ldots ,x_{i-1}\right),$$

where the conditional probability distributions are modeled by convolutions. Furthermore, the joint probability can be generalized to include exogenous series

$$p\left(x|h\right)=\prod_{i=1}^{t}p\left(x_{i}|x_{1},\ldots ,x_{i-1},h_{1},\ldots ,h_{i-1}\right),$$

where $h={\left(h_{t},h_{t-1},\ldots ,h_{1}\right)}^{T}$ is the exogenous series.

The WaveNet, as proposed by the authors, uses a gated activation unit on the output from the dilated convolution layer in Figure 2

$$z=tanh\left(w_{t,k}*x\right)⊙\sigma \left(w_{s,k}*x\right),$$

where * is a convolution operator, ⊙ is an element-wise multiplication error, σ is a sigmoid function, $w_{*,k}$ is the weights for the filters and *k* denotes the layer index. However, the model proposed in this paper will be restricted to only use a single activation function

(6) $$z=SeLU(w_{k}*x)$$

and the reason behind this is, again, that the gated activation function did not generalize well to the analyzed time series data.

When using an exogenous series to help improve the predictions, the authors introduce two different ways to condition the main series by the exogenous series. The first way, termed global conditioning, uses a conditional latent vector l (not dependent on time), accompanied with a filter $v_{k}$, and can be seen as a type of bias that influences the calculations across all timesteps

$$z=SeLU(w_{k}*x+v_{k}*l).$$

**Figure 3:**
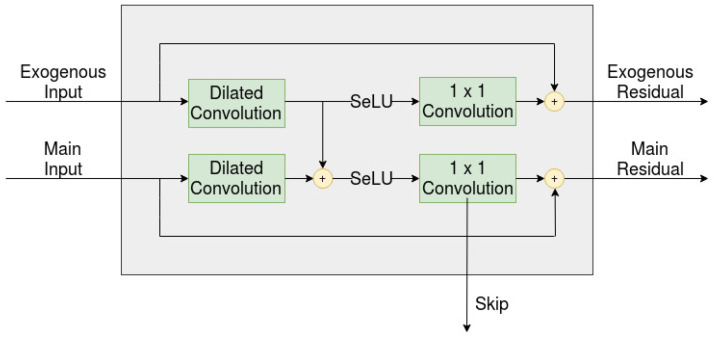
Overview of the residual layer, when the main series is conditioned by an exogenous series.

The other way, termed local conditioning, uses one or more conditional time series $h={\left(h_{t},h_{t-1},\ldots ,h_{1}\right)}^{T}$, that again influences the calculations across all timesteps

(7) $$z=SeLU(w_{k}*x+v_{k}*h)$$

and this is the approach that has been taken in this paper. This approach can further be observed in Figure 3.

Lastly, the WaveNet originally used a softmax activation function on the last output of the network, with the target values (raw audio) quantized into 256 different values. However, the softmax did not generalize well to the predictions for the financial time series used here, where the use of no activation function performed better when predicting the next day’s closing price of the S&P 500. In addition, when classifying the trend, the activation on the last output was seen as a hyperparameter, where the sigmoid and SeLU activations were compared against each other.

### 2.5. Walk-Forward Validation

Walk-forward validation, or walk-forward optimization, was suggested by Robert Pardo [17] and was brought forward since the ordinary cross-validation strategy is not well suited for time series data. The reason behind why cross-validation is not optimal for time series data is because temporal correlations exist in the data, and it should then be considered as “cheating” if one were to use future data points to predict past data points. This, most likely, leads to a lower training error, but should result in a higher validation/test error, i.e., it leads to poorer generalization due to overfitting. In order to avoid overfitting, the model should then, when making predictions at (or past) time *t*, only be trained on data points that were recorded before time *t*.

**Figure 4:**
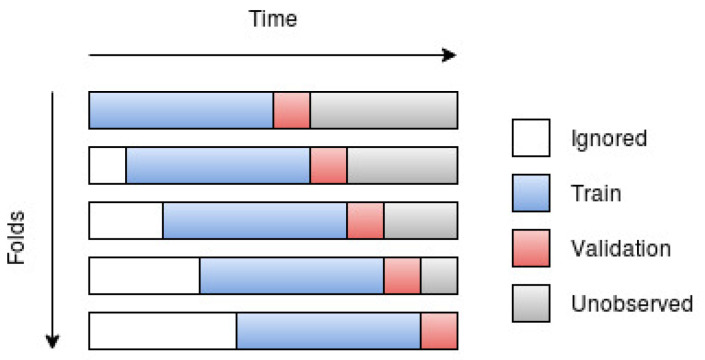
Walk-forward validation with five folds.

Depending on the data and the suggested model, one may choose between using all past observations (until the time of prediction) or using a fixed number of most recent observations as training data. The walk-forward scheme, using only a fixed number of observations, can be observed in Figure 4.

### 2.6. Efficient Market Hypothesis

Apart from the three sufficient assumptions, Eugen Fama (who can be seen as the father of modern EHM), lays out in [9] three different types of tests for the theory: weak form, where only past price movements are considered; semi-weak form, where other publicly available information is included, such as quarterly or annual reports; strong form, where some actors might have monopolistic access to relevant information. The tests done in this paper, outlined in the introduction, are clearly of the weak form.

Fama also brings to light three models that have historically been used to explain the movements of asset prices in an efficient market: the fair game model, the submartingale and the random walk. The fair game is by far the most general of the three, followed by the submartingale and then the random walk. However, this paper does not seek to explain the movements of the market, but merely to predict them, which means that any of the models can be used as the base model in the tests ahead.

Given the three assumptions on the market, the theory indicates that the best guess for any price in the future is the last known price (i.e., the best guess for tomorrow’s price of an asset is the price of that asset today). This can be altered to include a drift term, which can be determined by calculating the mean increment for a certain number of past observations, and the best guess then changes to be the last known price added with that mean increment.

## 3. Model Formulations and Methodology

### 3.1. Base Model

The base model in this paper, when predicting the next day’s closing price, was chosen to be that of a random walk, and this model can be modeled as an AR(1) process

$$X_{t}=c+\varphi_{1}X_{t-i}+\varepsilon_{t},$$

where $\varphi_{1}$ needs to equal one. $\varepsilon_{t}$ is here again a white noise, which accounts for the random fluctuations of the asset price. The *c* parameter is the drift of the random walk, and it can be determined by taking the mean of *k* previous increments

$$c=\frac{1}{k}\sum_{j=1}^{k}\left(X_{j}-X_{j-1}\right).$$

The best guess of the next day’s closing is obtained by taking the expectation of the random walk model ($\varphi_{1}$ equal to one)

(8) $$E\left(X_{t}\right)=E\left(c+X_{t-1}+\varepsilon_{t}\right)=c+X_{t-1},$$

which is the prediction that the base model used.

In the case of predicting the trend, the base model implemented a passive buy-and-hold strategy, which means that the model always predicts the trend to be up.

### 3.2. CNN Model

As stated in the introduction, a CNN model, inspired by the WaveNet, was compared to the base model, and two different approaches were needed in order to answer the research questions. The first approach was to structure the CNN as a univariate model, which only needed to be able to handle a single series (the series to make predictions over). This model can be expressed as a NAR model, which can be observed by studying Equation (2). Each element $x_{t}$ in the sequence is determined by a non-linear function *f* (the CNN in this case), which takes the past *p* elements in the series as input. The second approach was to structure the CNN as a multivariate model, which needed to be able to handle multiple series (the series to make predictions over, together with exogenous series). This model, on the other hand, can be expressed as a NARX model, which can be observed by studying Equation (3). Again, each $x_{t}$ is determined by a non-linear function *f*, which here takes the past *p* and *r* elements in the main and exogenous series as inputs.

The NAR and NARX models were here, for convenience, named the single- and multi-channel models. However, two different variants of the multi-channel model were tested in order to compare the different structures implemented in the original WaveNet paper and in [7,8].

As was mentioned in the introduction, the difference between the two variants is how the residuals from the exogenous series are handled. The implementation found in the WaveNet paper takes into account both the main series residuals as well as the exogenous series residuals, while the implementation in the second variant only takes into account the main series residuals. This can be visualized by observing Figure 3 and then ignoring the residual for each exogenous series. This, in turn, leads to each residual layer beyond the first layer having a similar structure to that of Figure 3. The two variants were named multi-channel-sparsely-connected model (multi-channel-sc) and multi-channel-fully-connected model (multi-channel-fc) in this paper. Furthermore, all models have adopted a dropout factor of 0.2 for all layers, since this leads to better performances for all three models.

**Figure 5:**
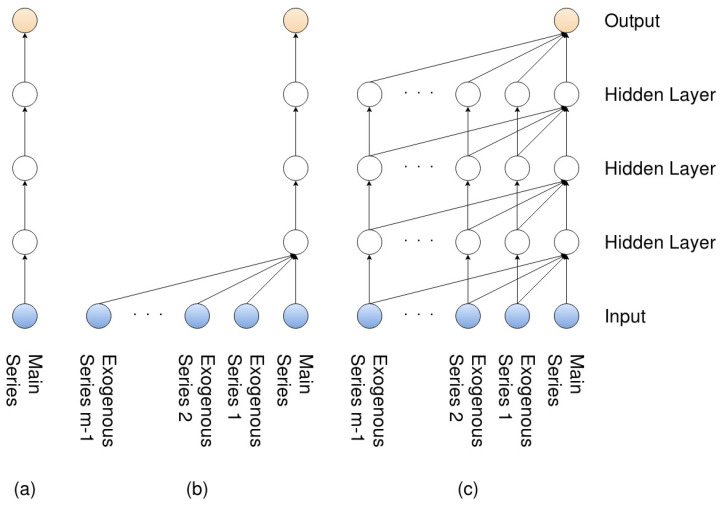
Side view of the dilated convolutional layers in Figure 1. (**a**) Only the main series is used; (**b**,**c**) when the main series is conditioned by an exogenous series, as in the model proposed in [7,8] and in the original WaveNet [5].

The three models (the single-channel as well the two variants of the multi-channel) can be further observed in Figure 5, which is a side view of the dilated layer shown in Figure 1. (a) represents the single-channel model, where no exogenous series are considered; therefore, the model only has to handle a single residual. (b) represents the multi-channel-sc model, where exogenous series are considered, but again, only a single residual is taken into account, while (c) represents the multi-channel-fc, where all residuals are taken into account.

### 3.3. Data Sampling and Structuring

The financial data, for the single-channel model as well as the more complex multi-channel models, were collected from Yahoo! Finance. The time interval between the observations was chosen to equal a single day, since the objective was to predict, for any given time, the next day’s closing price and the next day’s trend (i.e., the time series $x={\left(x_{t},\ldots ,x_{t-p}\right)}^{T}$, at any time *t*, was used to predict $x_{t+1}$ in the first case and $y_{t+1}$ in the second case, where $y_{t+1}=\left\{0,1\right\}$).

For the single-channel model, the series under consideration, at any time *t*, was $x={\left(x_{t},\ldots ,x_{t-p}\right)}^{T}$, which is composed of ordered closing prices from S&P 500. In the multi-channel models, different combinations of ordered OHLC (open, high, low and close) prices, of the S&P 500, VIX (implied volatility index of S&P 500), TY (10 year treasury note) and TYVIX (implied volatility index of TY) were considered. As mentioned before, the closing price of the S&P 500 was the main series, while the other series $Z=(z_{1},\ldots ,z_{m})$ were the exogenous series, where *i*, $z_{i}={\left(z_{i,t},\ldots ,z_{i,t-r}\right)}^{T}$, for every *i*. The values of *p* and *r* determine the orders of the NAR and NARX, and different values will be tested during the validation phase. However, only combinations where *p* and *r* are equal will be tested, and *p* will therefore be used to denote the length for both the main and exogenous series in the continuation of this paper.

The time span of the observations was chosen to be between the first day in 2010 and the last day in 2019, which resulted in 2515×m observations, where again *m* denotes the number of exogenous input series. Furthermore, since the models require *p* preceding observations, $x={\left(x_{t},\ldots ,x_{t-p}\right)}^{T}$, to make a prediction and then an additional observation, $x_{t+1}$, to evaluate this prediction, the number of time series that could be used for predictions were decreased to (2515−p−1)×m. These observations were then structured into time series, resulting in a tensor with dimension (2515−p−1)×p×m.

The resulting tensor was then divided into folds of equal size, which were used in order to implement the walk-forward scheme. The complete horizontal bars in Figure 4 should here be seen as the whole tensor, while the subsets consisting of the blue and red sections are the folds. The blue and red sections (training and test set of that particular fold) should be seen as a sliding window that “sweeps” across the complete set of time series. Whenever the model is done evaluating a certain fold, the window sweeps a specific number of steps (determined by the size of the test set) in time in order to evaluate the next fold.

By further observing Figure 4, it should become clear that the number of folds is influenced by the size of the training and test sets. The size of each fold could (and most likely should) be seen as a hyperparameter. However, due to the interest of time, the size of each fold was set to 250 series, which means that each fold had a dimension of 250×p×m. Each fold was then further divided into a training set (first 240 series) and a test set (last 10 series), where the test set was used to evaluate the model for that particular fold.

The sizes chosen for the training and test sets gave as a result 226 folds. These folds were then split in half, where the first half was used in order to validate the models (i.e., determine the most appropriate hyperparameters), and the second half was used to test the generalization of the optimal model found during the validation phase.

One last note about the data sampling is that when predicting the closing price, the time series were the original prices collected from Yahoo!, while when predicting the trend, the time series were changed to model the increments each day.

### 3.4. Validation and Backtesting

During the validation phase, different values for the length of the input series (i.e., the value of *p*), the number of residual connections (i.e., number of layers stacked upon each other, see Figure 1 and Figure 2) and the number of filters (explained in the theory section for CNNs) in each convolutional layer were considered. The values considered for *p* were 4, 6, 8 and 12, while the number of layers considered were 2 and 3, and the number of filters considered were 32, 64 and 96. When classifying the trend, the activation function applied to the last output was seen as a hyperparameter as well, and the sigmoid and SeLU activations were considered.

For the multi-channel models, all permutations of different combinations of the exogenous input series were considered. However, it was only for the mutli-channel-fc model that the exogenous series were seen as a hyperparameter. The optimal combination of exogenous series for the multi-channel-fc model was then chosen for the multi-channel-sc model as well. One final note regarding the hyperparameters is that the dilation rate was set to a fixed value equal to two, which is is the same rate as was proposed in the original WaveNet model, and the resulting dilated structure can be observed in Figure 1.

As was stated in the previous section, the validation was made on the first 113 folds. The overall mean for the error of these folds, for each combination of the hyperparameters above, was used in order to compare the different models, and the model with the lowest error was then used during the backtesting.

The batch size was set to equal one for all models, while the number of epochs was set to 300 in the single-channel model and 500 in the multi-channel models. The difference in epochs is here due to the added complexity that the exogenous series brings. An important note regarding the epochs and the evaluation of the models is that the model state, associated with the epoch with the lowest validation/test error, was ultimately chosen. This means that if a model made predictions over 300 epochs, but the lowest validation/test error was found during epoch 200, the model state (i.e., the value of the model’s weights) associated with epoch 200 were chosen as the best performing model for that particular fold.

### 3.5. Developing the Convolutional Neural Network

The networks were implemented using the Keras API, from the well known open-source library TensorFlow. Keras provides a range of different models to work with, where the most intuitive might be the Sequential model, where developers can add/stack layers, and then compile them into a network. However, the Sequential model does not provide enough freedom to construct the complexity introduced in the residual and skip part of the WaveNet. Keras functional API (more information regarding Keras functional API can be found on Keras official documentation https://keras.io/models/model/) might be less intuitive at first, but it does provide more freedom, since the order of the layers in the network is defined by having the output of every layer explicitly defined as an input parameter to the next layer in the network.

Furthermore, Keras comes with TensorFlow’s library of optimizers, which are used in order to estimate the parameters in the model and taken as an input parameter when compiling the model. The optimizer used here was the Adam optimizer, and the learning rate was set to equal 0.0001.

## 4. Results

### 4.1. Validation

Table 1 displays the validation error when predicting the next day’s closing price, while Table 2 displays the validation accuracy when classifying the trend. The *p* is again the length of the input time series, while *l* is the number of layers in the residual part of the network (see Figure 1), and *f* is the number of filters used in each convolutional layer. In Table 2, *a* denotes the used activation function.

The lowest validation error was achieved with *p*, *l* and *f* equal to 12, 3 and 96 for the single-channel model, while 8, 3, 64 and 8, 3, 32 were the optimal parameters for the multi-channel-sc model and the multi-channel-fc model, respectively. The highest validation accuracy was achieved with SeLU as the activation for all models and *p*, *l* and *f* equal to 12, 3 and 96 for the single-channel model, while 8, 3, 96 and 12, 3, 96 were the optimal parameters for the multi-channel-sc model and the multi-channel-fc model, respectively.

The validation error and validation accuracy for the multi-channel models are displayed only for the best combination of exogenous series found for the multi-fc-channel model, which proved to be just the highest daily value of the VIX in both cases.

### 4.2. Testing

**Figure 6:**
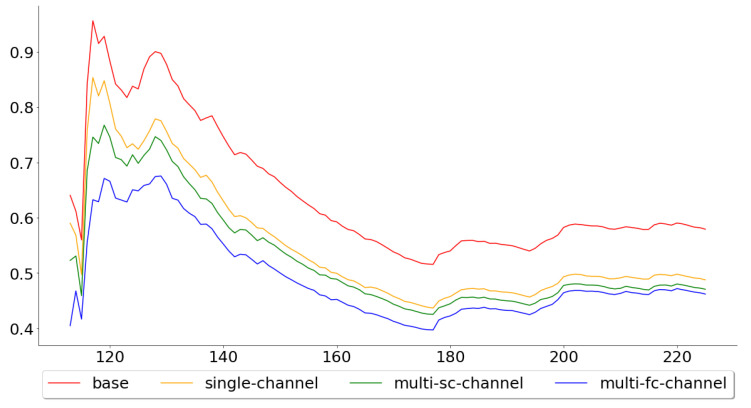
Cumulative mean of the MAPE for all 113 test folds.

**Figure 7:**
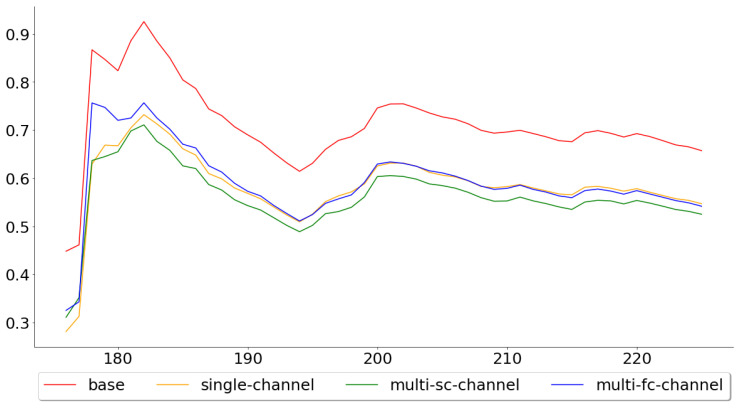
Cumulative mean of the MAPE for the last 50 test folds.

Figure 6 shows the cumulative mean, of the test error, for all 113 test folds, while Figure 7 shows the cumulative mean for the last 50. These two figures paint two different pictures of the single-channel and multi-channel models. The means across all 113 folds were 0.5793, 0.4877, 0.4707 and 0.4621, for the base, the single-channel, multi-channel-sc and multi-channel-fc models, respectively, while the means across the last 50 were 0.6572, 0.5468, 0.5250 and 0.5416. By looking at these numbers, one can see that the performance of the multi-channel-fc model to the base model, when predicting the next day’s closing price, was worse in the last 50 folds than for all 113 folds, while the reverse can be said about the single-channel and the multi-channel-sc models.

**Figure 8:**
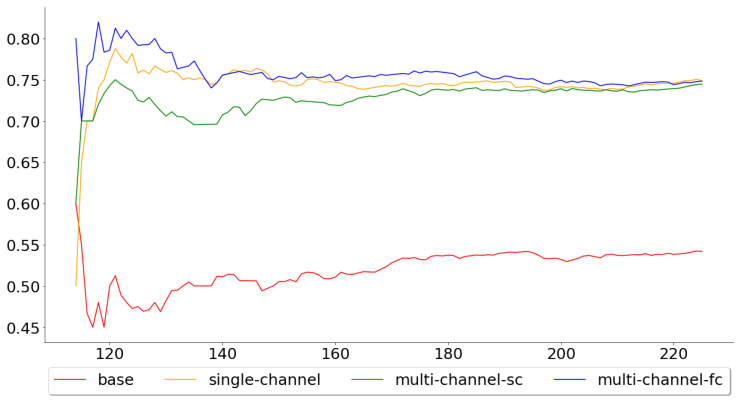
Cumulative mean for the accuracy for all 113 test folds.

**Figure 9:**
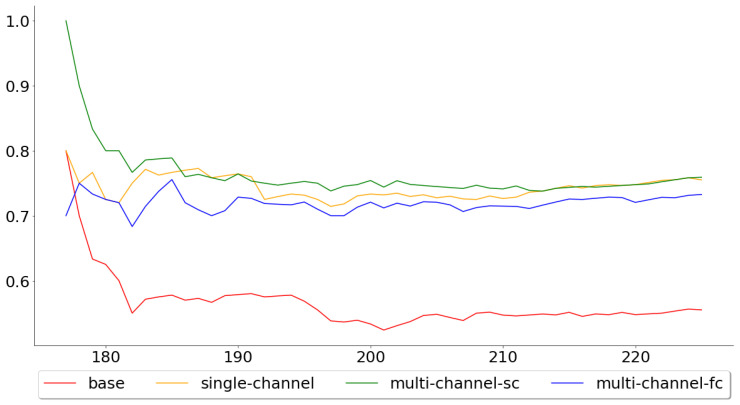
Cumulative mean for the accuracy for the last 50 test folds.

Figure 8 and Figure 9, on the other hand, show the cumulative mean of the validation error for all 113 test folds and the last 50 test folds. Again, the multi-channel-fc model started out well, but the performance compared to the single-channel and multi-channel-sc model worsened over time. The means across all 113 test folds were 0.5442, 0.7504, 0.7451 and 0.7496 for the base, single-channel, multi-channel-sc and multi-channel-fc models, respectively, while the means for the last 50 test folds were 0.5600, 0.7580, 0.7600 and 0.7360.

Both the single-channel and multi-channel models outperformed the base model over the test folds, which accounts for almost five years of observations. Furthermore, the multi-channel models clearly performed better than the single-channel model, when looking at the performance across all test folds. However, the positive effects of including the exogenous series seemed to wear off in the last folds for the complex multi-channel-fc model, while it actually increased for the simpler multi-channel-sc model. This suggests that the problem of generalization for the multi-channel-fc model probably lies in that the relationship between the main series and the exogenous series has been altered, which, interestingly enough, only affects the more complex model.

**Figure 10:**
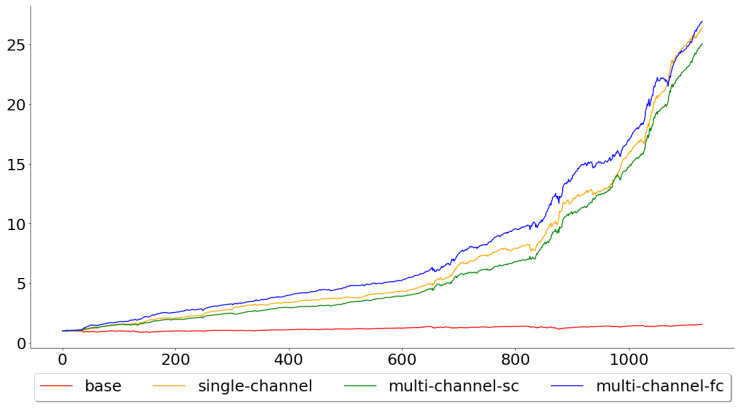
The calculated gain across all test folds, for all of the four models.

Figure 10 shows the gains for the models when applied to the test data. Both the single- and multi-channel models clearly outperformed the passive buy-and-hold strategy, which is to be expected, since the test accuracy for the base model was well below the other models. The gains were 1.5596, 26.3642, 25.0388 and 26.9360 for the base, single-channel, multi-channel-sc and multi-channel-fc, respectively, meaning that if one was to implement any of the WaveNet inspired models, during the specified period, he or she would have a profit of more than 24 times the original amount.

**Figure 11:**
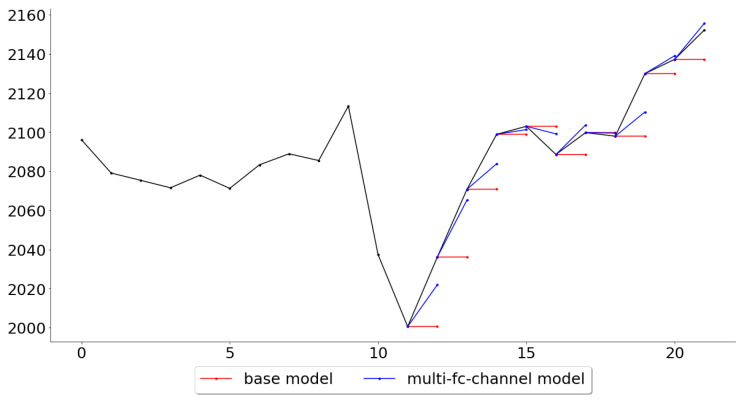
Predictions for the 10 test observations in test fold 25.

**Figure 12:**
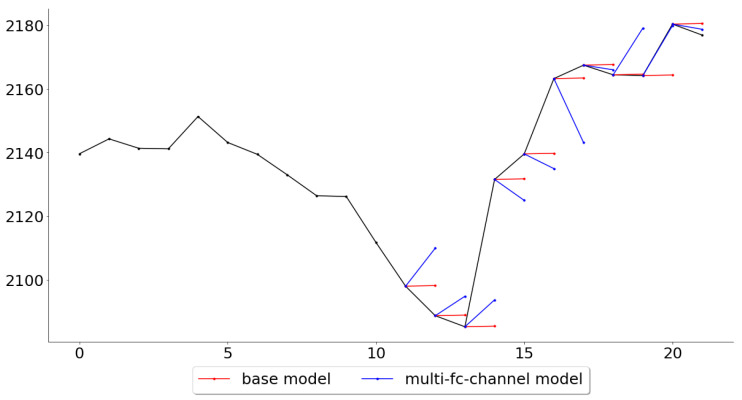
Predictions for the 10 test observations in test fold 34.

Lastly, while the multi-channel-fc model outperformed all other models across all folds, it is also of interest, for further work, to see in what settings the multivariate model performed the best and the worst. Figure 11 and Figure 12 give an example of these settings, where it shows the folds for which the multi-channel-fc model outperformed (fold 139) and underperformed (fold 148) the most against the base model.

## 5. Discussion

There is no real scientific basis for having the training size equal to 240 and validation/test size equal to 10, although it did perform better than having the sizes equal 950 and 50 respectively. It might seem odd to someone, with little or no experience in analyzing financial data, that one would choose to have a limit on the training size and why the models, evidently, perform better using fewer observations, since having a larger set of training observations is generally seen as a good thing. However, the financial markets are ever-changing, and the predictors (the past values in the time series) usually change with it. New paradigms find their way into the markets, while old paradigms may lose their impact over time. These paradigms can be imposed by certain events, such as an increase in monetary spending, the infamous Brexit or the current Covid-19 pandemic (especially the response, by the governments and central banks, to the pandemic). Paradigms can also be recurrent, such as the ones that are imposed by where we are in the short- and long-term debt cycle. Because of these shifts, developers are restricted in how far back in time they can look and, therefore, need to put restrictions on the training size.

The paper brings forward two very positive results, which are that the predictions for the next day’s closing price as well as the trend are made significantly better by the CNN models. However, the fact that the performance of the more complex multi-channel model decreases over time, against the other models, for both predicting the closing price and the trend, is indeed concerning. This became obvious when studying the change in performance between all 113 and the last 50 test folds. If both the multi-channel-sc and multi-channel-fc models performed worse in the last 50 folds, then it would have been easy to again “blame” the temporal structure of the financial data and, more specifically, the temporal dependencies between the main and exogenous series. However, only the more complex multi-channel model’s performance degraded, which means that the complexity (i.e., the intermingling between the series in all residual layers) is the primary issue. A solution might then be to have the complexity as a hyperparameter as well and not differentiate between the two structures, as was done here. In other words, the two models might more appropriately be seen as two extreme cases of the same model, in a similar way as having the number of filters set to 32 and 96 (see Table 1, 32 and 96 are the extreme cases for the number of filters). By looking at Figure 5 (with *p* equal to 16 in this case), one can see that the hyperparameter for the complexity has two more values to chose from (having the exogenous series to directly influence the second and third hidden layers). It would also be appropriate to compare the models against different time frames and asset classes to see if the less complex model indeed generalizes better over time, or if the result here was just a special case. However, viewing the complexity as a hyperparameter could prove to be beneficial in both cases.

The tests made in this paper were not primarily intended to judge the suitability of the models as trading systems, but rather if a deep learning approach could perform better than a very naive base model. However, the multi-channel models outperformed the base model by more than 20%, and this difference is quite significant and begs the question of what changes could be done in order for the model to be used as a trading system. While most of the predictions in fold 25, Figure 11, are indeed very accurate, the predictions in fold 34, Figure 12, would be disheartening for any trader to see if it were to be used as a trading system. This suggests that one should try to look for market conditions, or patterns, similar to the ones that were associated with low error rates in the training data. This could be done by clustering the time series, in an unsupervised manner, and then assign a score for each class represented by the clusters, where the score can be seen as the probability of the model to make good predictions during the conditions specific to that class. A condition classed in a cluster with a high score, such as the pattern in Figure 11, would probably prompt the trader to trust the system and to take a position (either long or short, depending on the prediction), while a condition classed in a cluster with a lower score would prompt the trader to stay out of the market or to follow another trading system that particular day.

## 6. Conclusions

The deep learning approach, inspired by the WaveNet structure, proved successful in extracting information from past price movements of the financial data. The result was a model that outperformed a naive base model by more than 20%, when predicting the next day’s closing price, and by more than 37% when predicting the next day’s trend.

The performance of the deep learning approach is most likely due to its exceptional ability to extract non-linear dependencies from the raw input data. However, as the field of deep learning applied to the financial market progresses, the predictive patterns found in the data might become increasingly hard to find. This would suggest that the fluctuations in the market would come to more and more mirror a system, where the only predictive power lies in the estimation of the forces acting on the objects, which are heavily influenced by the current sentiment in the market. A way to extract the sentiment, at any current moment, might be to analyze unstructured data, extracted from, for example, multiple news sources or social media feeds. Further study in text mining, applied to financial news sources, might therefore be merited and might be an area that will become increasingly important to the financial sector in the future.

## Figures and Tables

**Table 1:** Validation error for the different models, where *p* is the length of the time series, *l* is the number of residual layers and *f* is the number of filters.

Hyperparameters			MAPE		
p	l	f	Single-Channel	Multi-Channel-sc	Multi-Channel-fc
4	2	32	0.5787	0.5706	0.5599
4	2	64	0.5802	0.5527	0.5577
4	2	96	0.5783	0.5508	0.5587
6	2	32	0.5720	0.5491	0.5544
6	2	64	0.5752	0.5450	0.5426
6	2	96	0.5793	0.5462	0.5479
8	2	32	0.5764	0.5413	0.5498
8	2	64	0.5737	0.5445	0.5450
8	2	96	0.5796	0.5405	0.5495
8	3	32	0.5733	0.5518	**0.5251**
8	3	64	0.5692	**0.5259**	0.5377
8	3	96	0.5687	0.5468	0.5389
12	2	32	0.5714	0.5524	0.5526
12	2	64	0.5744	0.5478	0.5542
12	2	96	0.5744	0.5417	0.5422
12	3	32	0.5700	0.5298	0.5444
12	3	64	0.5672	0.5368	0.5290
12	3	96	**0.5584**	0.5312	0.5325

**Table 2:** Validation accuracy for the different models, where *a* is the activation function, *p* is the length of the time series, *l* is the number of residual layers and *f* is the number of filters.

Hyperparameters				Accuracy		
a	p	l	f	Single-Channel	Multi-Channel-sc	Multi-Channel-fc
Sigmoid	4	2	32	0.6035	0.6000	0.5779
Sigmoid	4	2	64	0.6150	0.5991	0.6283
Sigmoid	4	2	96	0.5956	0.6204	0.6637
Sigmoid	6	2	32	0.6283	0.6009	0.6283
Sigmoid	6	2	64	0.6115	0.6186	0.6319
Sigmoid	6	2	96	0.6097	0.6319	0.6389
Sigmoid	8	2	32	0.5938	0.6283	0.6146
Sigmoid	8	2	64	0.5956	0.6133	0.6248
Sigmoid	8	2	96	0.6248	0.5947	0.6363
Sigmoid	8	3	32	0.6150	0.6053	0.6000
Sigmoid	8	3	64	0.6265	0.6044	0.6451
Sigmoid	8	3	96	0.6487	0.6124	0.6327
Sigmoid	12	2	32	0.6009	0.6027	0.6115
Sigmoid	12	2	64	0.6035	0.6212	0.6327
Sigmoid	12	2	96	0.6168	0.5920	0.6398
Sigmoid	12	3	32	0.6168	0.5973	0.5973
Sigmoid	12	3	64	0.6195	0.6106	0.6248
Sigmoid	12	3	96	0.6442	0.6159	0.6292
SeLU	4	2	32	0.6752	0.6664	0.6894
SeLU	4	2	64	0.7053	0.7195	0.6920
SeLU	4	2	96	0.7018	0.7097	0.7469
SeLU	6	2	32	0.7062	0.6814	0.6938
SeLU	6	2	64	0.6947	0.7000	0.7133
SeLU	6	2	96	0.7204	0.7389	0.7230
SeLU	8	2	32	0.7018	0.6735	0.6717
SeLU	8	2	64	0.7071	0.6885	0.6841
SeLU	8	2	96	0.6947	0.7159	0.7257
SeLU	8	3	32	0.6549	0.6407	0.6619
SeLU	8	3	64	0.6973	0.7044	0.7133
SeLU	8	3	96	0.7336	**0.7345**	0.7425
SeLU	12	2	32	0.7097	0.6743	0.7018
SeLU	12	2	64	0.6752	0.7292	0.7027
SeLU	12	2	96	0.7434	0.7230	0.7142
SeLU	12	3	32	0.6699	0.6319	0.6637
SeLU	12	3	64	0.7257	0.7088	0.7434
SeLU	12	3	96	**0.7487**	0.7310	**0.7496**
